# Artificial Induction of Labor in Uræmia

**Published:** 1871-09

**Authors:** 


*Artificial Induction of Labor in Uræmia.* By SAMUEL C. BUSEY, M. D.

In this article, which is a reprint from the National Medical Journal, Dr. Busey considers two propositions. The first relates to the morality and safety of the operative procedure, and the second to the expediency of the proceeding in uræmic poisoning. The first was approved in 1756, and has been practiced ever since; the second is in the affirmative, on the ground, that the peril of the mother is diminished. Various comparative tables in this connection are added, thus making the paper more complete.

